# Labia majora lifting technique with polydioxanone threads

**DOI:** 10.1093/sexmed/qfaf064

**Published:** 2025-09-30

**Authors:** Savas O Aglamis, Selver K Akkaya, Elif O Sahin, Hanifi Sahin

**Affiliations:** Department of Obstetrics and Gynecology, Dr. Ozgur Aglamis Private Clinic, 34381, Sisli, Istanbul, Turkey; Department of Obstetrics and Gynecology, Dr. Selver Kubra Akkaya Private Clinic, 06690, Cankaya, Ankara, Turkey; Department of Obstetrics and Gynecology, Koru Ankara Hospital, 06690, Cankaya, Ankara, Turkey; Department of Obstetrics and Gynecology, Istün University, 34164, Gungoren, Istanbul, Turkey

**Keywords:** labio majors, labioplasty, genital esthetic

## Abstract

**Background:**

Polydioxanone (PDO) thread is a synthetic absorbable surgical suture used for rejuvenation and lifting.

**Aim:**

The aim of this study is to use PDO threads for rejuvenation and enlargement effect in patients with labium majus hypotrophy.

**Methods:**

Twenty-one patients with labia majora hypotrophy were included in the study. Conventionally, surgery, fat filling or hyaluronic acid filling is used for labia majora rejuvenation. In this study, a different technique, the PDO thread suspension technique, was applied. For PDO thread, Hyundae Meditech Co.Ltd's Secret Line Up product containing 50 mm screw thread with 30 G-38 mm needle tip was used. It was planned to use 10 PDO threads for right and left labia majora. After a total of 20 needles were inserted, the needles were removed one by one and the PDO threads remained in the subcutaneous superficial layer and the procedure was terminated 5 min later. Preoperative and postoperative the Female Genital Self-Image Scale (FGSIS) scores of the patients were compared.

**Outcomes:**

The overall FGSIS total score demonstrated a significant increase following the intervention.

**Results:**

The FGSIS total mean score in the preoperative period was increased in the postoperative period. This difference was statistically significant. Moreover, the mean score calculated for each parameter of FGSIS in the preoperative period increased significantly in the postoperative period.

**Clinical Implications:**

These findings indicate a favorable safety profile for the use of PDO threads in this clinical context.

**Strengths and Limitations:**

The strength of the study is to introduce a minimally invasive and effective method for labia majora lifting, on the other hand, the small number of patients in the study, limitation of the study.

**Conclusion:**

We would like to point out that in this study, we evaluated labium majus rejuvenation from the same perspective, based on the shaping and enlargement of genital appearance and its positive effect on self-confidence and increase in sexual functions. Unlike many labium majus rejuvenation procedures, this less invasive procedure has achieved similar results. In this context, it is a preferable alternative to surgery.

## Introduction

The number of surgical and non-surgical applications has increased by the increased popularity of genital esthetics and genital rejuvenation applications. According to plastic surgery statistics report by the American Plastic Surgery Association in 2019, the frequency of labiaplasty operations increased by 9% in 2019 compared to previous year.^1^ According to the American Society of Aesthetic Plastic Surgeons 2017 Statistics, the request for labiaplasty has increased by 217.2% from 2012 to 2017.^2^

Vaginal rejuvenation market growth is predicted to increase by 33.7% until 2026.^3^ Volume reduction, sagging, and wrinkles begin to appear on the skin of labium majus due to aging, rapid weight gain and loss, decrease in the amount of collagen, and slowing of hyaluronic acid production. Different treatment methods are used for labium majus rejuvenation to lighten the color and to give a brighter and younger skin and volume. These are topical medical applications, laser applications, radiofrequency applications, hyaluronic acid applications, and platelet rich plasma (PRP) applications.^3^ There are also surgical applications such as lipofilling applications, surgical flap, and dermal graft applications for augmentation and rejuvenation.^4^ Polydioxanone (PDO) thread is a synthetic absorbable surgical suture used for rejuvenation and lifting. It has been used for many years. Absorption occurs within 4-6 months. This reabsorption is carried out by hydrolysis, which triggers fibroblast production. As a result, collagen production increases in the applied area.^3^

The aim of the present study is to use PDO threads for rejuvenation and augmentation effect in patients with labium majus hypotrophy based on this effect. In this study, we aimed to achieve labia majora rejuvenation and lifting effect with a different technique, going beyond conventional methods.

## Methods

Twenty-one patients with labia majora hypotrophy such as sagging, volume loss, and wrinkles in the skin tissue of labium majus between 2021 and 2023 were included in the study. Patients undergoing labia majoraplasty, lipofilling, hyaluronic acid fillers or laser treatment before were excluded from the study. Written informed consent was obtained from all the participants. The present study was conducted in accordance with the standards of Good Clinical Practice (ICH-E6) and the principles of the Declaration of Helsinki.

### Surgical procedure

**Figure 1:**
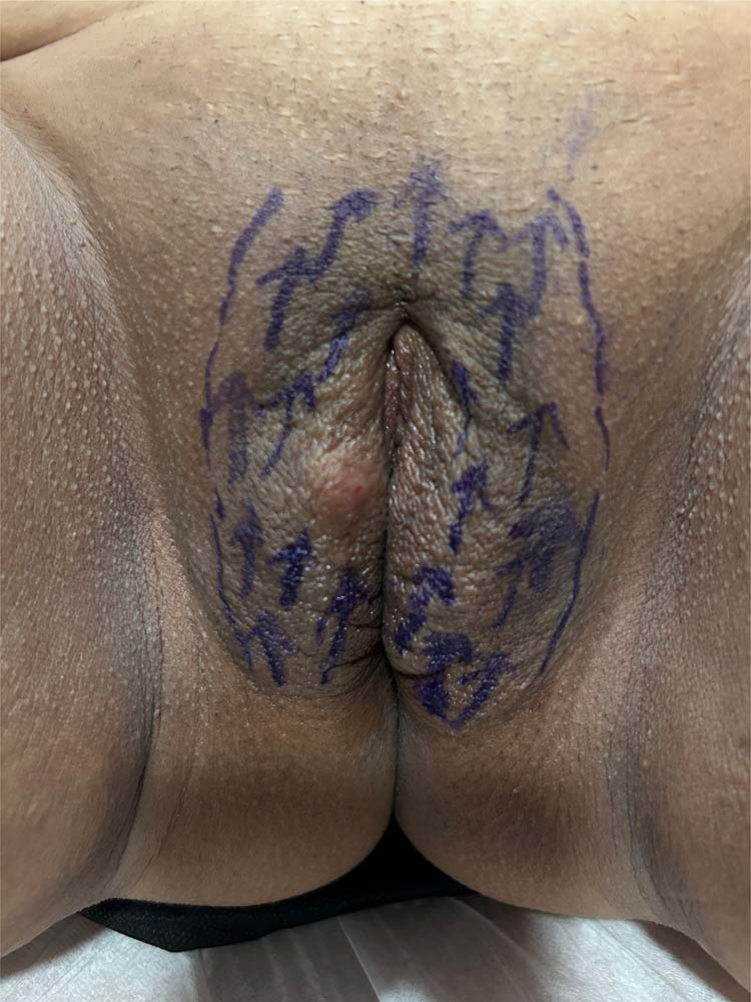
Determination of regions for Polydioxanone insertion.

**Figure 2:**
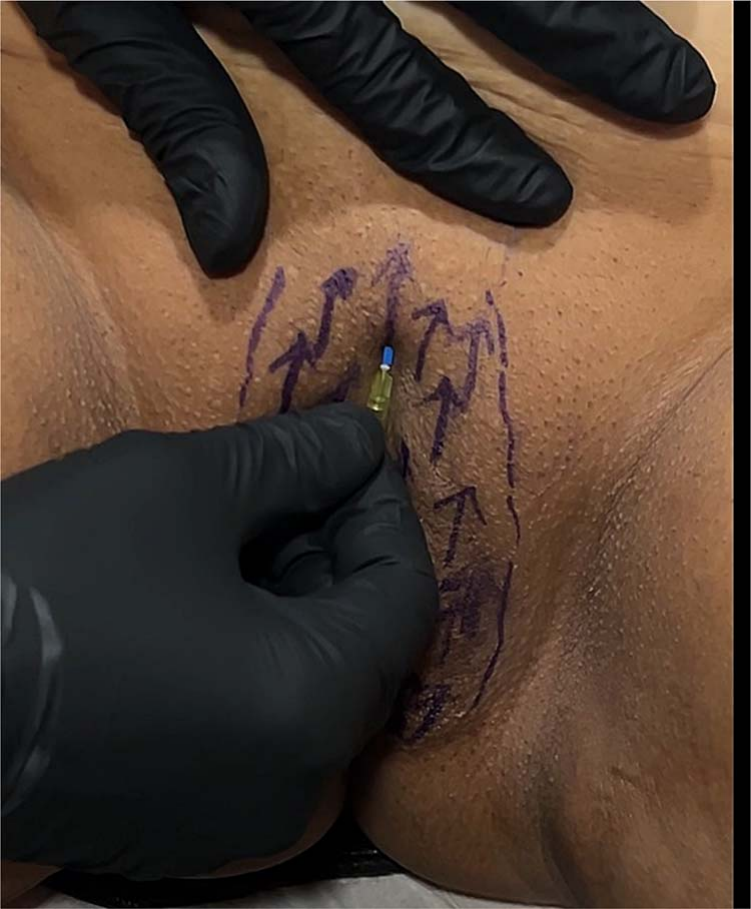
Polydioxanone insertion.

**Figure 3:**
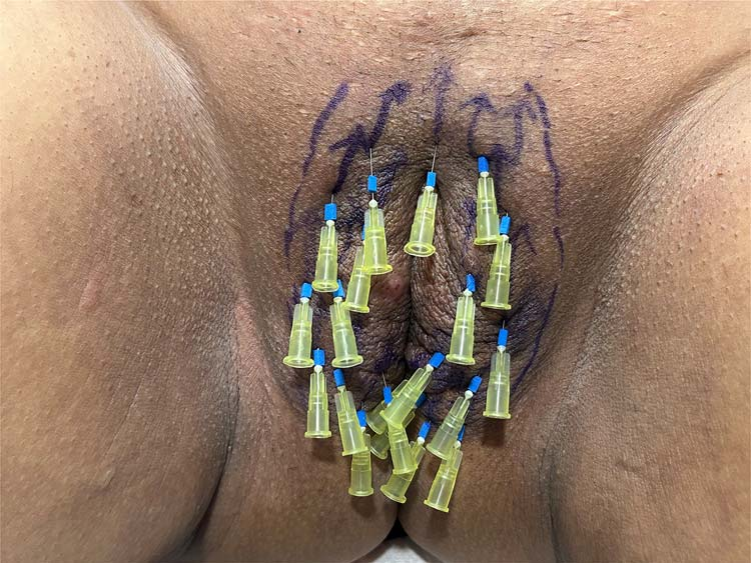
Insertion of twenty needles.

**Figure 4:**
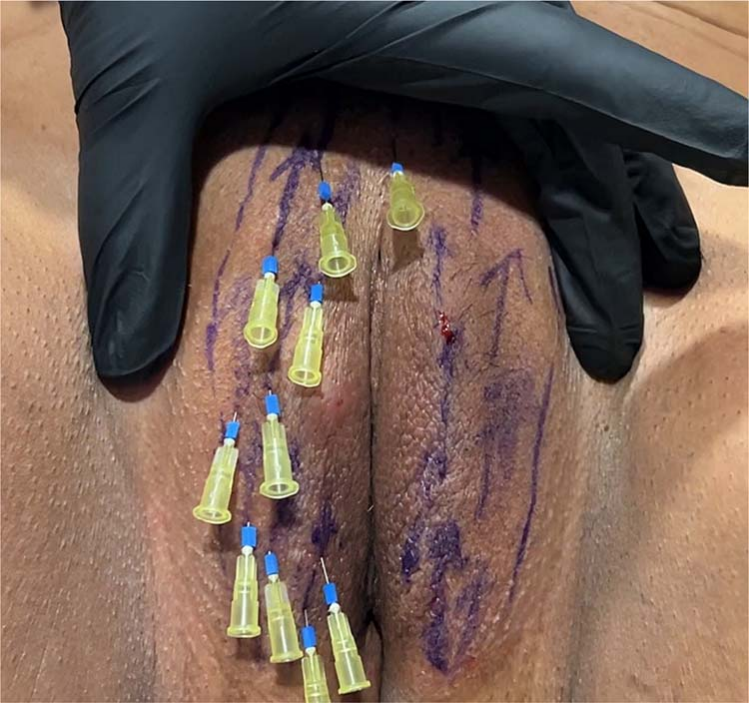
Removing of needles.

**Figure 5:**
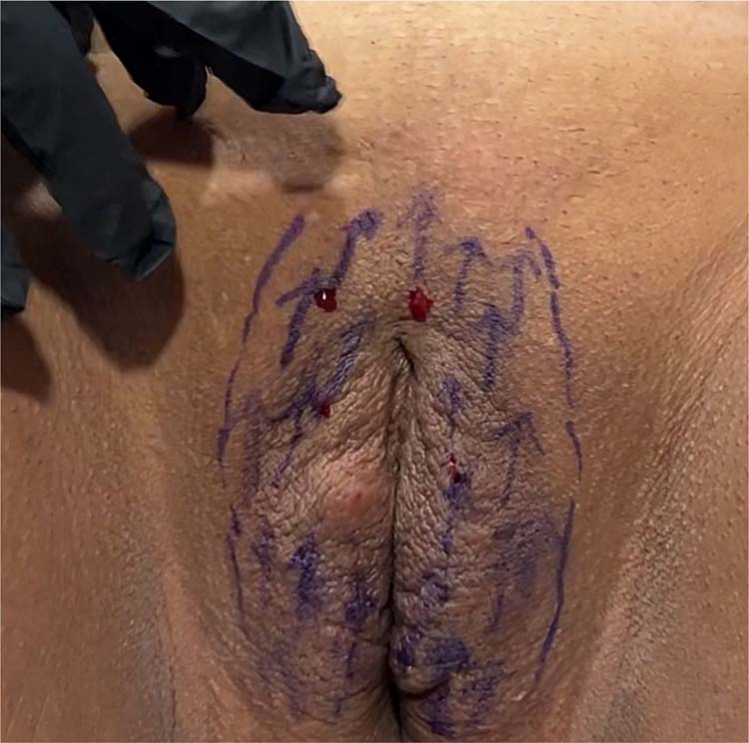
Termination of the procedure.

For the session 1, the patients were placed in the lithotomy position. The vulva was cleaned with chlorhexidine before the procedure. One cc of 1 ml local anesthetics (Jetocaine) containing 20 mg Lidocaine Hydrochloride and 0.0125 mg Epinephrine base was applied to each of both labia majora. Immediately afterwards, 30% (28% lidocaine-2% prilocaine) local anesthetic cream was applied to the vulva. After waiting for 10 min, the procedure was started. For PDO thread, Hyundae Meditech Co.Ltd's Secret Line Up product containing 50 mm screw thread with 30 G-38 mm needle tip was used. It was planned to use 10 PDO threads for right and left labia majora (Figure 1). The ends of the threads should be pointing upwards to the pubis. After the needle of the PDO thread inserted the skin at an angle of 90°, it was progressed superficially at an angle of 45° until the blue guide sponge of the needle touched the skin (Figure 2). After a total of 20 needles were inserted (Figure 3). The needles were removed one by one and the PDO threads remained in the subcutaneous superficial layer (Figure 4). After the procedure, moisturizing cream was applied to the treated area and the procedure was terminated 5 min later (Figure 5). The patients were discharged from the clinic half an hour after the procedure. It was ordered to use paracetamol 500 mg tablet 3 × 1 on the same day after the procedure. Hot shower, sauna, Turkish bath and sexual intercourse for 3 days were prohibited for the patients. The patients were invited back to the clinic 1 month later and the first procedure was applied in exactly the same way and the session 2 was completed. After the second session was completed, the patients were discharged from the clinic half an hour after the procedure. It was ordered to use paracetamol 500 mg tablet 3 × 1 on the same day after the procedure. The patients were prohibited from hot shower, sauna, Turkish bath and sexual intercourse for 3 days. The patients were examined in the clinic in the preoperative period and in the 1st month after the session 2 and evaluated with the Female Genital Self-Image Scale (FGSIS – Table 1). FGSIS is a seven-item questionnaire that has respondents rate each question on a 4-point response scale (strongly disagree [1 point], disagree [2 points], agree [3 points], or strongly agree [4 points]). An individual’s total score is obtained by adding the scores of individual questions and can range from 7 to 28. A higher score indicates a more positive genital self-image and significantly correlates with a women’s sexual function, sexual behavior, and their sexual and genital health-care behaviors.

**Table 1:** Female Genital Self-Image Scale.

FGSIS Original Version
I feel positively about my genitals I am satisfied with the appearance of my genitals I would feel comfortable letting a sexual partner look at my genitals I think my genitals smell fine I think my genitals work the way they are supposed to work I feel comfortable letting a health care provider examine my genitals I am not embarrassed about my genitals
Answers (1) Strongly disagree (2) Disagree (3) Agree (4) Strongly agree

**Figure 6:**
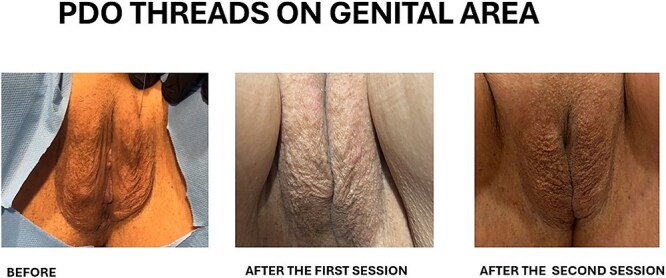
Images of preoperative and postoperative, a month after the 2nd session of the procedure.

Preoperative and postoperative, a month after the 2nd session of the procedure, images of the patient is Figure 6. Except for mild tenderness, superficial bruising or itching in the first 1-week period after the sessions 1 and 2, no side effects or complications were encountered.

### Statistical analysis

Coding and statistical analyses of the data were performed on computer using SPSS 22 software (IBM SPSS Statistics, IBM Corporation, Chicago, IL). Whether or not the variables were normally distributed was analyzed by Shapiro–Wilk tests. Variables were expressed as mean ± standard deviation. Paired Samples test and Wilcoxon test were used for the comparisons between dependent groups. *P*-value of <0.05 was considered as statistically significant.

## Results

A total of 21 female patients who underwent labia majora rejuvenation with PDO threads were included in the study. The mean age of the participants was 43.6 ± 4.4 years, and the mean body mass index was calculated as 26.8 ± 4.2, indicating that most of the patients were within the overweight category according to WHO classification. All participants completed both preoperative and postoperative assessments using the FGSIS.

The overall FGSIS total score demonstrated a significant increase following the intervention. The mean preoperative total score was 14.0 ± 3.5, which increased to 22.0 ± 3.1 in the postoperative evaluation. This improvement was found to be statistically highly significant (*P* < 0.001), indicating a notable enhancement in patients’ genital self-perception following PDO thread application.

In-depth analysis of each individual item of the FGSIS revealed consistent improvements across all parameters. The items measured various dimensions of female genital self-image, including emotional, visual, sexual, and functional perceptions (Table 2).

- Item 1 evaluated the extent to which the patient felt positively about her genitals. The preoperative mean score of 1.7 ± 0.7 significantly increased to 3.1 ± 0.7 postoperatively (*P* < 0.001).
- Item 2 assessed satisfaction with the appearance of the genitals. A significant improvement was observed, with the score rising from 1.7 ± 0.7 to 3.0 ± 0.7 (*P* < 0.001).
- Item 3 measured confidence during sexual activity in relation to genital appearance. This parameter improved from 1.7 ± 0.6 to 2.9 ± 0.7 (*P* < 0.001), suggesting a potential positive impact on sexual function or willingness to engage in intimacy.
- Item 4 focused on the perceived normalcy of genital appearance. This item showed a preoperative mean of 2.8 ± 0.8 and a postoperative mean of 3.5 ± 0.6 (*P* = 0.002), indicating that even patients who initially reported moderate satisfaction experienced further improvement.
- Item 5 addressed comfort in allowing a partner to view the genitals. The score rose significantly from 2.3 ± 0.8 to 3.3 ± 0.5 (*P* < 0.001), which may indicate increased confidence and reduced embarrassment.
- Item 6 assessed the patient’s perception of genital function as being normal. A marked improvement was observed, with the score increasing from 2.0 ± 0.8 to 3.2 ± 0.4 (*P* < 0.001).
- Item 7 evaluated how similar the patient believed her genitals were in appearance compared to others. This score improved from 1.9 ± 0.6 to 2.9 ± 0.5 (*P* < 0.001), suggesting reduced feelings of deviation or inferiority in comparison to perceived norms.

**Table 2:** Comparative analysis of preoperative and postoperative Female Genital Self-Image Scale scores of the patients who underwent labia majora rejuvenation with PDO.

**FGSIS parameters**	**Preoperative**	**Postoperative**	***P***
	Mean ± SD	Mean ± SD	
1	1.7 ± 0.7	3.1 ± 0.7	**<0.001**
2	1.7 ± 0.7	3 ± 0.7	**<0.001**
3	1.7 ± 0.6	2.9 ± 0.7	**<0.001**
4	2.8 ± 0.8	3.5 ± 0.6	**0.002**
5	2.3 ± 0.8	3.3 ± 0.5	**<0.001**
6	2 ± 0.8	3.2 ± 0.4	**<0.001**
7	1.9 ± 0.6	2.9 ± 0.5	**<0.001**
Total	14 ± 3.5	22 ± 3.1	**<0.001**

^*^Bold *P*-values indicate statistical significance.

A comprehensive comparison of the pre- and postoperative FGSIS scores, along with standard deviations and corresponding *P*-values, is presented in Table 1. All seven items showed statistically significant improvements, with six out of seven yielding *P*-values below 0.001. This supports the hypothesis that PDO thread-based labia majora rejuvenation has a positive and consistent effect on female genital self-image across multiple psychosocial and functional dimensions.

Throughout the study period, no adverse events, infections, thread extrusions, or procedural complications were reported. All patients tolerated the treatment well and completed the follow-up period without incident. These findings indicate a favorable safety profile for the use of PDO threads in this clinical context.

## Discussion

Cosmetic shaping and augmentation of labia majora may offer some functional benefits other than their appearance. Because according to the results of questionnaires conducted with patients, they have reported that they are more satisfied with sexual intercourse and appearance after the procedure.^5^ We would like to state that we evaluated rejuvenation of labium majus from the same point of view in this study, which we conducted in parallel with that study, based on the increase in sexual functions and the positive effect of genital appearance on self-confidence with shaping and augmentation. Moreover, both the correction of abnormalities and the beautification of the existing appearance in the genital area are demanded by modern women.^6^

The most commonly used surgical technique for labia majora rejuvenation is microfat and nanofat grafting.^7^ This is a surgical procedure and may bring along surgical care and some complications. Complications such as hematoma, ecchymosis, edema, and formation of palpable nodule may be encountered.^4^ We preferred to apply this method because the current study is a non-surgical application and will reduce the rate of encountering serious complications mentioned in previous surgical studies. Moreover, a surgical operating theater, surgical personnel, and pre- and post-operative care process necessary for a surgical procedure increase the cost of the procedure; whereas, the cost of the present study is low compared to all surgical procedures, which is considered as an advantage of the current study.

Another surgical procedure for labia majora rejuvenation and reshaping is labio majoraplasty. In other words, it is surgical removal of excess sagging tissue. In this technique, major bleeding after the procedure and even hematoma requiring additional surgery have been reported.^8^ The fact that the present study was minimally invasive compared to these studies seems to be an advantage of the technique. Moreover, even though small scars may be seen on the labium majus skin due to cannula insertion site or incision in both fat filling applications and surgeries, there was no visually noticeable scar related to a 30 G needle insertion site in the current study.

Another method used for labium majus rejuvenation and augmentation is hyaluronic acid filling. In a study, hyaluronic acid filling was carried out for labium majus rejuvenation and almost no serious complications were observed.^9^ The fact that no serious complication was observed in our technique is due to the fact that it is a minimally invasive procedure as in the present study. In their study, Kim *et al.*, performed 38- and 50-mm Gold Thread Implantation in the labia majora subcutaneous tissue under sedation anesthesia. They reported a significant improvement in laxity of labium majus after the procedure.^10^ Although gold is considered a safe material, complications related to gold implantation such as hypersensitivity reaction and local inflammatory reactions have been reported after upper-eyelid implant treatment.^11^ Since gold is an expensive product, the PDO threads we used in our treatment can be regarded as an advantage of our procedure because they provide a lower cost.

Poleva *et al.*, conducted a study by applying the absorbable thread material called Nano Spring 7, which is a 7-cm blunt guide, to the subcutaneous layer of the labia majora.^3^ In that study, they obtained cosmetically satisfactory results for patients similar to the current study. The fact that the needle was 3.5 cm in the current study makes our study more minimally invasive and advantageous compared to other studies with similar results.

PDO is a surgically absorbable suture material. It triggers fibroblast production in subcutaneous implantations and absorption occurs by hydrolysis within 6 months. By triggering the formation of type 1 and 3 collagen, subcutaneous connective tissue restructuring, augmentation and as a target result, contraction and lifting effect occur on the skin. Due to this feature, it can be used in many areas of the body, especially in facial rejuvenation applications.^12^

In a study, the formation of collagen around the PDO thread in patients was also proved by means of ultrasound images as well as the improvement in skin appearance and sagging evaluated by photographic control in patients.^13^

In the present study, based on the positive effects of PDO threads on the tissue, we applied PDO threads to the subcutaneous tissue of Labium majus for the first time as far as we have seen in the literature. The fact that we think that we will plan to provide labium majus rejuvenation, augmentation and lifting effect by exhibiting a minimally invasive approach can be seen as the advantage of the study. Moreover, the fact that PDO threads dissolve after 6 months and the total effect starts to decrease within 9 months can be seen as a disadvantage of the study.

The clinical application of PDO threads, traditionally used for tissue lifting, has demonstrated volumizing effects on the midface.^14^ In our study, the rejuvenation and augmentation effect of the labia majora is similar to this information.

In another study, PDO sutures were used to elevate the midface, and ease of use, simplicity and minimal complication rates were shown as advantages of the study.^15^ In our study, the fact that it is a simple and easy-to-apply technique, complications are minimal and not life-threatening can be shown as advantages of this technique over surgical techniques.

In another study, although the number of patients was very limited, a hybrid filler consisting of hyaluronic acid and calcium hydroxylapatite was used in addition to conventional hyaluronic acid fillers study.^16^

Nevertheless, the fact that it is a minimally invasive application and we use a cheaper suture material compared to other suture samples in office conditions with only local cream anesthesia can be considered as an advantage of the current study.

## Conclusion

PDO threads are a material that can be preferred in subcutaneous applications that have a rapid effect in the body, is safe and non-allergic, and have a duration of action of at least 6 months. It is widely used especially for face lifting purposes. Although thread suspension methods with other materials have been used in the vaginal area, although rare, PDO sutures have not been used before. Our positive results with PDO threads in labium majus tissue can be seen as a sign that the interest in minimally invasive approaches in cosmetic gynecology will increase more. Further investigation with a larger number of patients should be conducted to confirm data of the present study.

## References

[1] Surgeons, A.S.o.P . National Clearinghouse of Plastic Surgery Procedural Statistics. Plastic Surgery Statistics Report 2019; 2020. Available from: https://www.plasticsurgery.org/news/plastic-surgery-statistics?sub=2019+Plastic+Surgery+Statistics.

[2] Willis RN, Szymanski KD, Patel BC. In: Rhett N, ed. Labiaplasty Minora Reduction. Treasure Island (FL): StatPearls; 2024: ineligible companies.

[3] Poleva I, Markova N, Sulamanidze M. Open pilot study on the rejuvenation effect of absorbable threads in the genital area. Clin Cosmet Investig Dermatol. 2023;16:2237–2248. 10.2147/CCID.S416232 PMC10440063 37605787

[4] Jabbour S, Kechichian E, Hersant B, et al. Labia Majora augmentation: a systematic review of the literature. Aesthet Surg J. 2017;37(10):1157–1164. 10.1093/asj/sjx056 28449124

[5] Felicio Y de A . Labial surgery. Aesthet Surg J. 2007;27(3):322–328. 10.1016/j.asj.2007.03.003 19341661

[6] Cohen PR . Genital rejuvenation: the next frontier in medical and cosmetic dermatology. Dermatol Online J. 2018;24(9):1–3. 10.5070/D3249041410 30677826

[7] Menkes S, SidAhmed-Mezi M, Meningaud JP, Benadiba L, Magalon G, Hersant B. Microfat and Nanofat grafting in genital rejuvenation. Aesthet Surg J. 2021;41(9):1060–1067. 10.1093/asj/sjaa118 32386063

[8] Di Saia JP . An unusual staged labial rejuvenation. J Sex Med. 2008;5(5):1263–1267. 10.1111/j.1743-6109.2008.00802.x 18331252

[9] Fasola E, Gazzola R. Labia Majora augmentation with hyaluronic acid filler: technique and results. Aesthet Surg J. 2016;36(10):1155–1163. 10.1093/asj/sjw083 27241363

[10] Kim SM, Won YS, Kim SK. Gold thread implantation for female sexual dysfunction and vaginal laxity: a preliminary investigation. J Menopausal Med. 2020;26(2):130–134. 10.6118/jmm.19024 32893514 PMC7475290

[11] Kilduff CLS, Casswell EJ, Imonikhe R, Marjanovic B. Type IV hypersensitivity to gold weight upper-eyelid implant: case report and review of the literature. Ocul Immunol Inflamm. 2018;26(6):910–914. 10.1080/09273948.2017.1311922 28471252

[12] Cobo R . Use of Polydioxanone threads as an alternative in nonsurgical procedures in facial rejuvenation. Facial Plastic Surg. 2020;36(04):447–452. 10.1055/s-0040-1714266 32866981

[13] Lots TCC . Effect of pdo facelift threads on facial skin tissues: an ultrasonographic analysis. J Cosmet Dermatol. 2023;22(9):2534–2541. 10.1111/jocd.15761 37128828

[14] Yi KH, Park SY. Volumizing threads (Jamber) in the midface and managing side effects: clinical cases. Arch Plast Surg. 2024;51(04):350–355. 10.1055/a-2303-5156 39034977 PMC11257737

[15] Pan C, Liu Z, Liu K. Lower blepharoplasty with mid-face elevation: a Polydioxanone (PDO) barbed suture loop for lid-cheek junction blending. Aesth Plast Surg. 2024;48(16):3082–3090. 10.1007/s00266-024-04133-8 38806827

[16] Acevedo A, Parra AM, Amado AM, et al. Labia Majora rejuvenation with hybrid filler: a narrative review of the literature and report of two cases. J Cosmet Dermatol. 2025;24(3):e70074. 10.1111/jocd.70074 40013509 PMC11866467

